# Americans’ support for future pandemic policies: insights from a national survey

**DOI:** 10.1093/haschl/qxae171

**Published:** 2024-12-10

**Authors:** Gillian K SteelFisher, Mary G Findling, Hannah L Caporello, Jazmyne Sutton, Emma Dewhurst, Katherine Evans, Brian C Castrucci

**Affiliations:** Department of Health Policy and Management, Harvard T.H. Chan School of Public Health, Boston, MA 02115, United States; Department of Health Policy and Management, Harvard T.H. Chan School of Public Health, Boston, MA 02115, United States; Department of Health Policy and Management, Harvard T.H. Chan School of Public Health, Boston, MA 02115, United States; SSRS, Glen Mills, PA 19342, United States; The de Beaumont Foundation, Bethesda, MD 20814, United States; The de Beaumont Foundation, Bethesda, MD 20814, United States; The de Beaumont Foundation, Bethesda, MD 20814, United States

**Keywords:** pandemic, infectious disease outbreaks, public opinion, survey, vaccination, masks, politics, policy, COVID-19, H5N1, polarization

## Abstract

The arrival of bird flu (H5N1) is a poignant reminder of the need for public health leaders to understand Americans’ evolving perspectives on pandemic mitigation policies. To guide response efforts, we conducted a nationally representative opinion survey among 1017 U.S. adults in 2024. Majorities said they would be likely to support each of 4 policies in a future pandemic scenario (related to masking requirements, school closures, restaurant closures, and healthcare worker vaccination requirements). About half (49%) were likely to support all 4 policies, while 32% expressed mixed support. Support varied by gender, age, race, ethnicity, income, metropolitan and parental status, political party, and COVID-specific comorbidities. Roughly 80% expressed concern that future pandemic policies would hurt the economy, be based on political or pharmaceutical company/business interests, pander to critics, or further polarize society. Results suggest public support for future pandemic policies may be wider than media reports suggest, though important divisions exist and concerns about design and implementation are widespread. The most appealing policies will explicitly consider economic impacts and target populations at risk during clear time frames, with scope for personal choice. Ensuring that policies are made without undue political or commercial influence will remain a central challenge for public health leaders.

## Teaser

The arrival of bird flu (H5N1) in the United States less than a year after the official end of the COVID-19 pandemic is a poignant reminder of the ongoing threat posed by emerging infectious diseases and pandemics. To create an effective response that will engage the public when needed, it is critical that public health leaders understand evolving public support for pandemic policies. However, there is little rigorous research about how public opinion may have shifted in the wake of the pandemic. This study provides an updated view of public perceptions that can guide public health leaders in pandemic planning and response after COVID-19.

## Introduction

The arrival of bird flu (H5N1) in the United States less than a year after the official end of the COVID-19 pandemic is a poignant reminder of the ongoing threat posed by emerging infectious diseases and pandemics. To create an effective response that will engage the public when needed, it is critical that public health leaders understand evolving public support for pandemic policies.^1-5^ However, public opinion polling that was common during COVID has all but stopped, and there is no peer-reviewed literature examining public views of mitigation policies since the pandemic ended.^2^ Without research, media stories become leading sources of information to describe public sentiment. However, media coverage of where the public stands on pandemic policy has had important limitations.^6-8^ Fundamentally, media rely on current polls and therefore cannot cover sentiment about future policies. Moreover, news stories often focus on segments of the population with more extreme views using election polls of voters or stories without polls at all—for example, stories about specific people like actors within the anti-vaccination movement.^6-14^ While important from a news perspective, such stories can collectively create an overemphasis on division, without much coverage of middle-ground views or shared perspectives across subsets of the population.^9,15^ In turn, this creates risk for public health leaders who rely on media coverage for understanding public sentiment when they plan policies, programs, and communication.

This study provides an updated and more complete view of public perceptions to guide public health leaders in pandemic planning and response. We examined support for policies related to masking, indoor dining closures, school closures, and vaccination. We also examined concerns about those policies that could help guide policy development and communication. Finally, we analyzed differences in views among segments of the public, including those with shared sociodemographic characteristics, health risk factors, and ideological standpoints, including religiosity and partisanship.

## Data and methods

### Study population and survey design

Data come from an online and telephone survey using a nationally representative, probability-based sample of 1017 US adults, aged 18 years and older. This survey was designed and analyzed by researchers from Harvard T.H. Chan School of Public Health (HSPH), with input from de Beaumont Foundation staff. The study was determined to be exempt by the HSPH Institutional Review Board. Details about methods, question wording, weighting, analyses, and robustness checks are available as Supplementary material.

### Data collection

Data were collected from March 21 to April 3, 2024, using the SSRS opinion panel, a high-quality, nationally representative, probability-based survey panel.^16^ Recruitment methods included address-based sampling and random digit-dialing. Though most participants completed this survey online, a small segment completed it via telephone to ensure the sample includes adults who do not regularly use the internet. Questions were administered in English and Spanish.

### Survey instrument and measures

We developed the questionnaire using the American Association of Public Opinion Research’s best practices for survey research and followed their reporting guidelines.^17^ The questionnaire was developed after reviewing relevant prior opinion research and was assessed for bias, balance, and comprehension using pretests.^2,3,18-22^

The survey instrument included questions about participants’ support for 4 policies on masking, healthcare worker vaccination, school closures, and indoor dining closures in a future COVID-like pandemic scenario. Question wording was intended to elicit broader attitudes about these policies and to reduce focus on specific policy details. Therefore, we asked about the likelihood of supporting each *type* of policy in the future. Likelihood was measured using a 4-point Likert scale. Respondents were then asked about their levels of concern (also using a 4-point scale) regarding future policy risks, using a list of 11 items derived from prior research.^3,18,19^

### Statistical analysis

Data were weighted by the probability of recruitment and selection, census region, population density, gender, race and ethnicity, education, age, internet use, and civic engagement (see Supplementary material).^23,24^ Among adults invited to participate, 44% completed the survey. This completion rate reflects the survey’s rapid-response design, which is parallel to studies conducted during COVID and other outbreaks.^19,25^ Research suggests data from rapid-response surveys are comparable to higher-response surveys after weighting.^26,27^ Demographic differences between this sample and national sources were <1% after weighting.

We analyzed support for each policy using a 4-point Likert scale. We created a composite measure of support using participants’ responses across the 4 policies, grouping respondents into 3 categories: (1) those likely (“very” or “somewhat”) to support all policies; (2) those with mixed support across policies (likely to support 1, 2, or 3 policies); and (3) those not likely (“not at all” or “not too” likely) to support any policies. We also measured concerns about policy risks using a 4-point Likert scale and analyzed the fraction who were concerned (“very” and “somewhat”) about each.

We used 2-tailed *t-*tests to compare bivariate differences in the likelihood of support for policies (individually and as a composite measure) as well as policy concerns by gender, age, having comorbidities associated with severe illness from COVID-19, parent/guardian status, race and ethnicity, household income, metropolitan status, education, political party affiliation, and religious service attendance. Differences below *P* < 0.05 were considered statistically significant.

We conducted sensitivity analyses and robustness checks using alternative summary metrics and ordinal logistic regression. All analyses were performed in Stata, version 15.0, with weighted data.

### Limitations

Study limitations include those common in probability-based surveys, such as the use of self-reported data and potential non-response bias beyond what was addressed with weighting. Social desirability bias may be a risk if respondents felt indirect pressure to support or oppose pandemic policies or to minimize or exaggerate their concerns. Data are cross-sectional and therefore cannot prove causality. Further, these results reflect views of broad policies in a hypothetical scenario, and public views of specific policies may change depending on the details of future outbreaks or policies.

## Results

### Support for future pandemic policies

Majorities of adults surveyed said they would be likely to support each of the 4 policies in the future, COVID-like pandemic scenario: mask requirements in stores/businesses for 2-3 months (71% likely to support, including 52% “very” and 18% “somewhat” likely), public school closures for a couple of weeks (68% likely to support, including 43% “very” and 25% “somewhat”), restaurant and bar indoor seating closures for a couple of weeks (67% likely to support, including 43% “very” and 24% “somewhat”), and healthcare worker vaccination requirements (64% likely to support, including 45% “very” and 19% “somewhat”) (Table 1). Some patterns in support were evident across subgroups: Black and Hispanic/Latino adults, lower-income adults, and Democrats and Independents were more likely to support each of the policies compared with their counterparts.

**Table 1.** Public support for 4 policies in a future pandemic scenario like COVID-19, overall and by select sociodemographic, political, and health characteristics, March-April 2024.

	“Very” or “somewhat” likely to support public health agency recommendations that ______ in a future pandemic scenario				Across all 4 policies future pandemic policies:			
Characteristic	People be required to wear masks in stores and businesses for 2-3 months when transmission gets high	Public schools close for a couple of weeks when transmission gets high	Restaurants/bars be required to close indoor seating or move seating outdoors for a couple of weeks when transmission gets high	Healthcare workers be required to get a vaccine because the hospitals are getting overwhelmed	ANY SUPPORT Very/somewhat likely to support ANY of the 4 policies	HIGH SUPPORT Very/somewhat likely to support all 4 policies	MIXED SUPPORT Very/somewhat likely to support 1-3 policies	NO SUPPORT Not very/somewhat likely to support ANY of the 4 policies
Total *n* = 1017	71	68	67	64	81	49	32	19
Gender								
Men (a) *n* = 494	66	65	62	63	77	47	30	23^b^
Women (b) *n* = 519	75^a^	71	72^a^	63	84^a^	51	34	16
Age								
18-34 (c) *n* = 246	75	70	69	67	88^e^	45	43^def^	12
35-44 (d) *n* = 178	66	67	69	60	78	49	29	22^c^
45-64 (e) *n* = 315	68	67	62	59	76	48	28	24^c^
65+ (f) *n* = 278	72	69	69	69	80	55	26	20
COVID-19 comorbidities								
Yes (g) *n* = 367	78^h^	77^h^	74^h^	68	87^h^	57^h^	30	13
No (h) *n* = 643	67	64	64	61	78	45	33	22^g^
Parent/Guardian of Child <18 during COVID								
Yes (i) *n* = 325	66	64	63	53	75	42	33	25^j^
No (j) *n* = 688	73	70	68	69^i^	83^i^	52^i^	32	17
Race and Ethnicity								
White (k) *n* = 599	61	60	59	56	74	40	34	26^lm^
Black (l) *n* = 158	87^k^	84^k^	82^k^	72^k^	93^k^	62^k^	31	7
Hispanic/Latino (m) *n* = 183	86^k^	81^k^	79^k^	76^k^	91^k^	60^k^	32	9
Annual household income								
<$50 000 (n) *n* = 453	81^op^	76^op^	72^p^	69^o^	87^op^	53^p^	34	13
$50 000-<$100 000 (o) *n* = 305	65	66	67	58	77	48	29	23^n^
$100 000+ (p) *n* = 248	60	56	58	59	75	41	34	25^n^
Metropolitan Status								
Urban (q) *n* = 323	76^s^	70	72	72^rs^	87^s^	52	35	13
Suburban (r) *n* = 556	70	68	66	61	80	49	31	20
Rural (s) *n* = 130	62	65	60	53	72	41	31	28^q^
Education								
HS or Less (t) *n* = 389	72	72	69	63	82	49	33	18
Some Coll (u) *n* = 272	65	66	60	58	77	42	35	23
College + (v) *n* = 356	73	66	70	68	83	53^u^	29	17
Political party affiliation								
Republican/leaners (w) *n* = 432	50	51	47	41	64	27	37^y^	36^yx^
Independent (x) *n* = 178	74^w^	68^w^	66^w^	65^w^	86^w^	45^w^	41^y^	14^y^
Democrat/leaners (y) *n* = 406	93^wx^	88^wx^	91^wx^	90^wx^	98^wx^	76^wx^	22	2
Religiosity (attendance)								
Currently attends religious services in-person (z) *n* = 360	68	67	65	57	80	43	36	20
Does not currently attend religious services (A) *n* = 652	72	68	68	67^z^	81	51	30	19

Data are from a 2024 nationally representative, probability-based online and telephone survey of 1017 US adults aged 18 years or older. Weighted percentages are displayed. Percentages may not add up to 100 due to rounding. Full question wording is available online as Supplementary material. Analyses were conducted using two-tailed *t-*tests. ^a–A^Value significantly higher than comparison group/row at *P* < 0.05. For race/ethnicity, statistical comparisons were only made between non-White vs White participants. Respondents with other racial/ethnic identities and other gender identities are included in the total but not shown due to low sample size. Parent/Guardian of Child <18 denotes parents/guardians whose children were living in their household during COVID. Independent includes politically unaffiliated adults. Religiosity denotes that adults have attended religious services in person in the last month.

When looking across policies, only 19% opposed all 4. By contrast, half (49%) were likely to support all 4 policies, and 32% had mixed support, meaning that 81% overall were likely to support at least 1 (Table 1). Majorities of adults in every subgroup said they were likely to support at least 1 policy, including higher-income adults earning $100 000+/year (75%), those in rural areas (72%), and Republicans/leaners (64%). Still, attitudes varied substantially among subgroups. Women, younger adults (ages 18-34), those with COVID-19 comorbidities, non-parents, Black and Hispanic/Latino adults, lower-income adults, those in urban areas, and Democrats and Independents were all more likely to support at least 1 policy compared with counterparts. Differences by COVID-19 comorbidities, parental status, Hispanic/Latino ethnicity, and political party remained robust in controlled comparisons using ordinal logistic regression (see Supplementary Material, Table A3).

### Concerns about future pandemic policies

**Figure 1.**
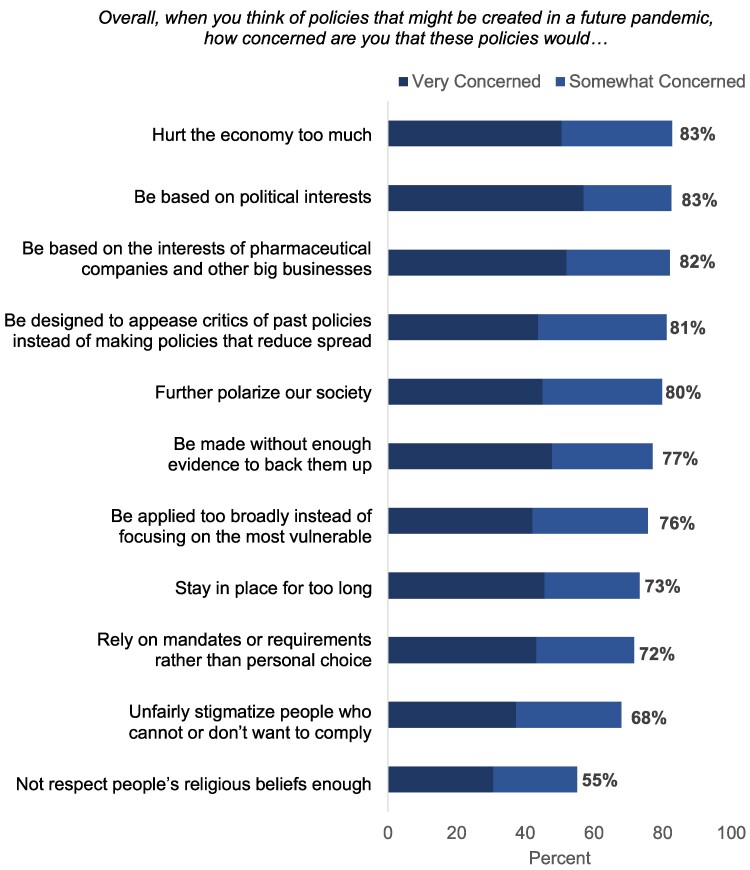
U.S. adults’ concerns about future pandemic policies, March-April 2024. Data are from a 2024 nationally representative, probability-based online and telephone survey of 1017 US adults aged 18 years or older. Weighted percentages are displayed. Items ranked by % of adults who were concerned (“very” or “somewhat”) about each item. Percentages may not add up to 100% due to rounding, and don’t know/refused responses are included in the total but are not displayed.

When asked about the risks of policies in a future pandemic, a majority of adults were concerned about all 11 issues (Figure 1). Roughly 4 in 5 were concerned that future pandemic policies would: hurt the economy too much (83% concerned, including 51% “very” and 32% “somewhat”), be based on political interests (83% concerned, including 57% “very” and 25% “somewhat”), be based on pharmaceutical companies and other big business interests (82% concerned, including 52% “very” and 30% “somewhat”), be designed to appease critics of past policies (81% concerned, including 44% “very” and 37% “somewhat”), or further polarize society (80% concerned, including 45% “very” and 35% “somewhat”). Additionally, roughly 3 in 4 were concerned about risks that policies would be created with insufficient evidence, could be applied too widely or too long, or would rely on mandates rather than choice.

There were relatively few large differences in concern levels among subgroups, given an apparent ceiling effect when majorities in nearly every sociodemographic subgroup said they were concerned about every issue listed (Supplementary Material, Table A5). Still, there were patterns of smaller differences. For example, Republicans were consistently more concerned about future policy risks than Democrats, showing more concern across 10 of the 11 risks. Religious adults were somewhat consistently more concerned than non-religious adults, showing more concern across 7 of the 11 risks, and those without college degrees showed more concern than those with college degrees across 6 risks.

## Discussion

Findings from this national survey study suggest that after COVID-19, there is sizable public support for future pandemic policy approaches, though support is far from absolute. Importantly, there is strong support for policy approaches across diverse areas of life where mitigation may be needed, including businesses, restaurants, hospitals, and schools. Further, support extends across an array of tools, including those based on individual behaviors like masking and vaccination, which have often been portrayed as having less widespread support by the media.^10,28^ Majority support for at least some policy action also cuts across every segment of the population we examined, including groups less commonly supportive of pandemic mitigation measures such as Republicans, those with less education, those living in rural areas, and those who are more religious.^1,2,4,29-32^ Divisions still exist, and in particular, Republicans remain consistently less supportive of future pandemic policies than Democrats or Independents. However, results overall suggest more appeal among Republicans and other groups than has generally been characterized in the lay press. Future research might examine the intersection between additional predictors of policy support, including ideological beliefs and trust in policy institutions, to examine how views continue to evolve.^33^

At the same time, findings point to specific features of pandemic policies that invoke concern even among supporters.^2,4^ This suggests that future policy support may hinge on the details and risks, which is consistent with long-standing research about public views of policy development but can be overlooked in practice.^34^ Risks drawing the most widespread concern include those with very practical implications, like negative economic impacts, as well as risks that government would not base policies on epidemiological evidence and instead they would be chaped by politics, commercial interests, or public pandering. Also drawing high concern are risks focused on scope, including worries that policies will be too broad or too long-lasting. Finally, there are concerns about societal implications, including that people will face discrimination or that the policies themselves will further divisions. While there are differences in the levels of concern along now-expected sociodemographic lines, including party and religious background, it is remarkable that concerns are so widespread across different segments of the public.

## Conclusion

After COVID, there is a narrow path for public health leaders to garner public support for pandemic policies in the future. The most appealing policies will be those targeting populations most at risk during clear time frames and emphasizing personal choice. Explicitly discussing the epidemiological logic of policies while acknowledging any economic implications and additional risks will be helpful. Moreover, making policies without undue influence from political or commercial spheres will remain a central challenge. As leaders communicate recommendations to the public, they will need to remain vigilant about partisan interpretations of their approaches. There is more receptivity from the public across political parties than leaders might think, but they must find partners to help connect with groups cast as unreceptive to public health measures.^1-3,29,31^ Further communications research is also needed to help avoid framing messages to trigger partisan interpretations.^1^ Finally, ongoing examination of public opinion in response to infectious illness and specific policy approaches will be essential. Together, these steps will help ensure that all Americans’ benefit from the protections of public health in future outbreaks.

## Disclaimer

The findings and conclusions in this report are those of the authors and do not necessarily represent the official position of the de Beaumont Foundation, HSPH, or SSRS.

## Supplementary Material

qxae171_Supplementary_Data
