# Supplementary material for: Americans’ support for future pandemic policies: insights from a national survey
Source: Health Aff Sch. 2024 Dec 10;2(12):qxae171. doi: 10.1093/haschl/qxae171 (PMC11683585; doi:10.1093/haschl/qxae171)
Supplement: qxae171_Supplementary_Data [file qxae171_supplementary_data.zip › Final Supplement Dec 18 2024.docx]

Supplemental Online Content

Table of Contents

[A) eMethods - Questionnaire Development and Question Language 2](#_Toc184637517)

[B) eMethods - Sampling, Weighting, and Margin of Sampling Error Details 3](#_Toc184637518)

[C) eMethods – Table A1. Comparison Between Sample and National Benchmarks for Key Demographics 4](#_Toc184637519)

[D) Supplementary Results - Table A2. Full Likert Scale Results of U.S. Adults’ Reporting Likelihood of Supporting Future Pandemic Policy Approaches 5](#_Toc184637520)

[E) Supplementary Results - Table A3. Ordinal logistic regression model, displaying adjusted odds ratios of support across future pandemic policies (95% CI) 7](#_Toc184637521)

[F) Supplementary Results - Table A4. Full Likert Scale Results of U.S. Adults’ Concerns about Eleven Risks of Future Pandemic Policies 8](#_Toc184637522)

[G) Supplementary Results - Table A5. Percentage of U.S. Adults’ Concerns (% “Very” or “Somewhat”) about Eleven Risks of Future Pandemic Policies 9](#_Toc184637523)

This supplemental material has been provided by the authors to give readers additional information about their work.

# A) eMethods - Questionnaire Development and Question Language

**Details on Questionnaire Development**

The questionnaire was developed using American Association of Public Opinion Research *Best Practices for Survey Research*.^[[1]](#footnote-1)^ The content and wording of questions, response options, and question order and flow were all developed after reviewing prior surveys. Before fielding, the questionnaire was reviewed for bias, balance, and comprehension, with pretesting among a small of participants to improve the survey quality.

| **Questions** | | | **Response Categories** |
| --- | --- | --- | --- |
| **COVID-19 Policies [Respondents were asked about Policies 1 – 4 in a randomized order]** | | |  |
| **[Policy 1 – School Closures]**  Imagine for a minute that, in the next few years, there were a pandemic caused by a new respiratory virus that was similar to COVID-19 and that public health agencies recommended that public schools close for a couple of weeks when transmission gets high. Children would have classes online during that time where possible. How likely would you be to support this kind of policy? | | | Very likely  Somewhat likely  Not too likely  Not at all likely |
| **[Policy 2 – Masks]**  Imagine for a minute that, in the next few years, there were a pandemic caused by a new respiratory virus that was similar to COVID-19 and that public health agencies recommended that people be required to wear masks in stores and businesses for 2-3 months when transmission gets high. How likely would you be to support this kind of policy? | | | Very likely  Somewhat likely  Not too likely  Not at all likely |
| **[Policy 3 – Indoor Restaurant/Bar Closures]**  Imagine for a minute that in the next few years, there were a pandemic caused by a new respiratory virus that was similar to COVID-19 and that public health agencies recommended that restaurants and bars be required to close indoor seating or move seating outdoors for a couple of weeks when transmission gets high. How likely would you be to support this kind of policy? | | | Very likely  Somewhat likely  Not too likely  Not at all likely |
| **[Policy 4 – Healthcare Worker Vaccination Requirements]**  Imagine for a minute that in the next few years, there were a pandemic caused by a new respiratory virus that was similar to COVID-19 and that public health agencies recommended that healthcare workers be required to get a vaccine because the hospitals are getting overwhelmed. How likely would you be to support this kind of policy? | | | Very likely  Somewhat likely  Not too likely  Not at all likely |
| **Concerns** | | |  |
| Overall, when you think of policies that might be created in a future pandemic, how concerned are you that these policies would…  …Be based on political interests  …Be based on the interests of pharmaceutical companies and other big businesses  …Hurt the economy too much  …Be made without enough evidence to back them up  …Stay in place for too long  …Further polarize our society  …Be designed to appease critics of past policies instead of making policies that reduce spread  …Rely on mandates or requirements rather than personal choice  …Be applied too broadly instead of focusing on the most vulnerable  …Unfairly stigmatize people who cannot or don’t want to comply  …Not respect people’s religious beliefs enough | | | Very concerned  Somewhat concerned  Not too concerned  Not at all concerned |
| **COVID-19 Comorbidities** | | |  |
| Have you been told by a doctor or health professional that you have any of the following medical conditions: A serious heart, lung, kidney, or brain condition; a mental health condition; substance use disorder; cancer; diabetes; obesity or overweight; sickle-cell disease; tuberculosis; or decreased immunity? | | | Yes  No |
| **Parent/Guardian of Child <18** | | |  |
| Between March 2020 and May 2023, were you the parent or guardian of any children who were under the age of 18 and living in your household? | | | Yes  No |
| **Political Party Affiliation** | | |  |
| In politics today, do you consider yourself a: (Republican), (Democrat), an Independent, or something else?  (Follow-up among those who are not Republican or Democrat): Do you LEAN more towards the Republican Party or the Democratic Party? | | Republican/Lean Republican  Democrat/Lean Democrat  Independent/Don’t lean toward either party (unaffiliated) | |
| **Religious Service Attendance** | | |  |
| In the last month, did you attend religious services in person at a church, synagogue, mosque or other house of worship? | Yes, attended in the last month  No, did not attend in the last month | | |

# B) eMethods - Sampling, Weighting, and Margin of Sampling Error Details

Respondents were reached through the SSRS Opinion Panel,^[[2]](#footnote-2)^ a nationally representative probability-based web panel. Given that this is a probability-based panel, findings are statistically projectable to the general U.S. adult population. A total of 957 surveys were completed by panelists online, and 60 interviews were completed by phone with panelists who do not have access to the internet, or who do not use it. The survey was administered from March 21 – April 3, 2024.

The sample was weighted in stages. The first stage of the weighting was the application of a base weight to account for different selection probabilities and response rates across sample strata. In the second stage, sample demographics were post-stratified to match population parameters. Data were weighted by gender, race and ethnicity, education, age, and geographically by Census region and population density. Data were also weighted by additional metrics standard among many internet wave panels to reduce nonresponse bias – internet use and, civic engagement – because civically engaged respondents and higher-frequency internet users differ from the general population and are over-represented among panelists.^[[3]](#footnote-3)^ Comparisons between the survey sample and national benchmarks for key demographics are shown in the next section. The design effect for this survey was 1.82, and the margin of error for the entire sample is ±4.1 percentage points at the 95% confidence interval.

The participation rate for this survey was expected due to the rapid response nature of fielding the survey. Prior research suggests that the resulting data are comparable to longer-term, higher-response surveys when reweighted to key population parameters.^[[4]](#footnote-4)^ After weighting, differences between the survey sample and national sources for all demographic characteristics were less than 1 percentage point (see Table A1 below).

# C) eMethods – Table A1. Comparison Between Sample and National Benchmarks for Key Demographics

| Variable |  | Benchmark % | Weighted % |
| --- | --- | --- | --- |
| Gender | Male | 49.0% | 48.7% |
|  | Female | 51.0% | 51.3% |
| Age (in years) | 18-24 | 11.8% | 11.6% |
|  | 25-34 | 17.4% | 17.3% |
|  | 35-44 | 16.9% | 17.1% |
|  | 45-64 | 31.5% | 31.4% |
|  | 65+ | 22.4% | 22.5% |
| Education | HS or less | 38.2% | 38.2% |
|  | Some College | 26.4% | 26.5% |
|  | College+ | 35.4% | 35.4% |
| Region | North East | 17.4% | 17.6% |
|  | Midwest | 20.5% | 20.3% |
|  | South | 38.6% | 38.5% |
|  | West | 23.6% | 23.5% |
| Civic Engagement | Engaged | 27.0% | 27.5% |
|  | Not engaged | 73.0% | 72.5% |
| Race and Ethnicity | African American/Black, Non-Hispanic/Latinx | 12.1% | 12.0% |
|  | Asian, Non-Hispanic/Latinx | 6.5% | 6.6% |
|  | Hispanic/Latinx, Native Born | 8.4% | 8.6% |
|  | Hispanic/ Latinx, Foreign Born | 9.1% | 8.7% |
|  | White, Non-Hispanic/Latinx | 61.3% | 61.5% |
|  | Other, Non-Hispanic/Latinx | 2.6% | 2.5% |
| Density Quintiles | 1 | 20.0% | 20.2% |
|  | 2 | 20.0% | 19.8% |
|  | 3 | 20.0% | 19.8% |
|  | 4 | 20.0% | 20.1% |
|  | 5 | 20.0% | 20.2% |
| Internet Use Frequency | Almost constantly | 42.0% | 42.6% |
|  | Several times a day | 44.1% | 44.2% |
|  | Less often/Not an Internet User | 13.9% | 13.2% |

Table A1 shows key demographics of the sample compared to benchmark data.

- Gender, age, education, race and ethnicity, and region benchmarks were derived from 2023 Current Population Survey (CPS).^[[5]](#footnote-5)^ In the survey, gender age, education, and race and ethnicity were self-reported by participants.
- The civic engagement benchmark was derived from the September 2021 Volunteering and Civic Life Supplement data^[[6]](#footnote-6)^ of the Current Population Survey (CPS).
- The population density came from Claritas Pop-Facts Premier 2023.^[[7]](#footnote-7)^
- The internet frequency benchmark is from the National Public Opinion Reference Survey (NPORS) for Pew Research Center - May 19 to Sept 5, 2023.^[[8]](#footnote-8)^

Weights were trimmed to prevent individual interviews from having too much influence on final results.

As shown in Table A1, differences between the weighted sample and benchmarks were all less than 1 percentage point.

# D) Supplementary Results - Table A2. Full Likert Scale Results of U.S. Adults’ Reporting Likelihood of Supporting Future Pandemic Policy Approaches

* indicate values <.5%

Imagine for a minute that, in the next few years, there were a pandemic caused by a new respiratory virus that was similar to COVID-19 and that public health agencies recommended that people be required to wear masks in stores and businesses for 2-3 months when transmission gets high. How likely would you be to support this kind of policy?

|  | % |
| --- | --- |
| **Very/Somewhat likely (NET)** | **71** |
| Very likely | 52 |
| Somewhat likely | 18 |
| **Not too/Not at all likely (NET)** | **29** |
| Not too likely | 12 |
| Not at all likely | 17 |
| **Don’t know/Refused/Web blank** | ***** |

Imagine for a minute that in the next few years, there were a pandemic caused by a new respiratory virus that was similar to COVID-19 and that public health agencies recommended that restaurants and bars be required to close indoor seating or move seating outdoors for a couple of weeks when transmission gets high. How likely would you be to support this kind of policy?

|  | % |
| --- | --- |
| **Very/Somewhat likely (NET)** | **67** |
| Very likely | 43 |
| Somewhat likely | 24 |
| **Not too/Not at all likely (NET)** | **33** |
| Not too likely | 16 |
| Not at all likely | 17 |
| **Don’t know/Refused/Web blank** | ***** |

Imagine for a minute that, in the next few years, there were a pandemic caused by a new respiratory virus that was similar to COVID-19 and that public health agencies recommended that public schools close for a couple of weeks when transmission gets high. Children would have classes online during that time where possible. How likely would you be to support this kind of policy?

|  | % |
| --- | --- |
| **Very/Somewhat likely (NET)** | **68** |
| Very likely | 43 |
| Somewhat likely | 25 |
| **Not too/Not at all likely (NET)** | **32** |
| Not too likely | 18 |
| Not at all likely | 14 |
| **Don’t know/Refused/Web blank** | ***** |

Imagine for a minute that in the next few years, there were a pandemic caused by a new respiratory virus that was similar to COVID-19 and that public health agencies recommended that healthcare workers be required to get a vaccine because the hospitals are getting overwhelmed. How likely would you be to support this kind of policy?

|  | % |
| --- | --- |
| **Very/Somewhat likely (NET)** | **64** |
| Very likely | 45 |
| Somewhat likely | 19 |
| **Not too/Not at all likely (NET)** | **36** |
| Not too likely | 15 |
| Not at all likely | 21 |
| **Don’t know/Refused/Web blank** | ***** |

# E) Supplementary Results - Table A3. Ordinal logistic regression model, displaying adjusted odds ratios of support across future pandemic policies (95% CI)

| **Ordinal logistic regression model, displaying adjusted odds ratios of support across future pandemic policies (95% CI) (n=990 US adults)** | | |
| --- | --- | --- |
| Gender | |  |
| Men | | 1 [Reference] |
| Women | | 0.94 (0.65, 1.36) |
| Age | |  |
| 18-34 | | 1 [Reference] |
| 35-44 | | 1.17 (0.66, 2.06) |
| 45-64 | | 0.89 (0.56, 1.41) |
| 65+ | | 1.04 (0.61, 1.75) |
| COVID-19 comorbidities | |  |
| Yes | | **1.81* (1.23, 2.68)** |
| No | | 1 [Reference] |
| Parent/Guardian of Child <18 | |  |
| Yes | | 1 [Reference] |
| No | | **1.65* (1.08, 2.50)** |
| Race/Ethnicity | |  |
| White | | 1 [Reference] |
| Black | | 1.41 (0.86, 2.32) |
| Hispanic/Latino | | **2.67* (1.02, 7.01)** |
| Annual household income | |  |
| <$50,000 | | 1.50 (0.92, 2.43) |
| $50,000-<$100,000 | | 1.06 (0.65, 1.72) |
| $100,000+ | | 1 [Reference] |
| Metropolitan Status | |  |
| Urban | | 0.93 (0.53, 1.61) |
| Suburban | | 1.11, 0.69, 1.81) |
| Rural | | 1 [Reference] |
| Education | |  |
| HS or Less | | 1 [Reference] |
| Some Coll | | 1.09 (0.69, 1.73) |
| College+ | | 1.45 (0.92, 2.29) |
| Political Party Affiliation | |  |
|  | Republican/leaner | 1 [Reference] |
| Independent/unaffiliated | | **2.08* (1.30, 3.32)** |
| Democrat/leaner | | **8.90* (5.82, 13.62)** |

Table notes: Odds ratios with 95% Confidence Intervals are displayed. These results use an ordinal logistic regression model to estimate the adjusted odds of adults indicating higher levels of support for future pandemic policies, using the three-point policy support composite measure specified in Table 1 of the article as the outcome (likely to support all four policy measures vs. likely to support between one and three policy measures vs. not likely to support any policy measures). *Significant at p<0.05.

Data come from a 2024 nationally representative online and telephone survey of 1,017 US adults ages 18 and older, with 990 adults included in the final sample for modeling. Sample sizes vary across models because don’t know/refused responses were coded as missing. In addition, adults identifying as “other” for race/ethnicity and as “non-binary” or “other” for gender were included in models but results are not reported, due to low sample size. Based on Wald tests conducted to determine whether each variable significantly contributed to each model, we did not include religious service attendance in final models.

# F) Supplementary Results - Table A4. Full Likert Scale Results of U.S. Adults’ Concerns about Eleven Risks of Future Pandemic Policies

Question: Overall, when you think of policies that might be created in a future pandemic, how concerned are you that these policies would… [randomized]

|  | **Very/ Somewhat concerned (NET)** | **Very concerned** | **Somewhat concerned** | **Not too concerned** | **Not at all concerned** |
| --- | --- | --- | --- | --- | --- |
| a. Rely on mandates or requirements rather than personal choice | 72 | 43 | 28 | 17 | 11 |
| b. Stay in place for too long | 73 | 46 | 28 | 19 | 7 |
| c. Be made without enough evidence to back them up | 77 | 48 | 29 | 18 | 5 |
| d. Not respect people’s religious beliefs enough | 55 | 31 | 24 | 23 | 22 |
| e. Hurt the economy too much | 83 | 51 | 32 | 12 | 5 |
| f. Be applied too broadly instead of focusing on the most vulnerable | 76 | 42 | 34 | 18 | 6 |
| g. Be based on political interests | 83 | 57 | 25 | 12 | 5 |
| h. Be based on the interests of pharmaceutical companies and other big businesses | 82 | 52 | 30 | 11 | 7 |
| i. Further polarize our society | 80 | 45 | 35 | 13 | 7 |
| j. Be designed to appease critics of past policies instead of making policies that reduce spread | 81 | 44 | 37 | 13 | 6 |
| k. Unfairly stigmatize people who cannot or don’t want to comply | 68 | 37 | 31 | 18 | 14 |

# G) Supplementary Results - Table A5. Percentage of U.S. Adults’ Concerns (% “Very” or “Somewhat”) about Eleven Risks of Future Pandemic Policies

|  |  | % “very” or “somewhat” concerned that future pandemic policies would… | | | | | | | |  |  |
| --- | --- | --- | --- | --- | --- | --- | --- | --- | --- | --- | --- |
|  | Hurt the economy too much | Be based on political interests | Be based on pharmaceutical/ business interests | Be designed to appease critics of past policies | Further polarize our society | Be made without enough evidence to back them up | Be applied too broadly instead of focusing on the most vulnerable | Stay in place for too long | Rely on mandates or requirements rather than personal choice | Unfairly stigmatize people who cannot or don’t want to comply | Not respect people’s religious beliefs enough |
| Total | 83 | 83 | 82 | 81 | 80 | 77 | 76 | 73 | 72 | 68 | 55 |
| Gender |  |  |  |  |  |  |  |  |  |  |  |
| Men (a) n=494 | 78 | 83 | 80 | 81 | 80 | 79 | 73 | 73 | 72 | 66 | 55 |
| Women (b) n=519 | 87^a^ | 83 | 84 | 82 | 80 | 75 | 79 | 74 | 71 | 69 | 55 |
| Age |  |  |  |  |  |  |  |  |  |  |  |
| 18-34 (c) n=246 | 81 | 78 | 82 | 79 | 77 | 74 | 74 | 75 | 70 | 68 | 50 |
| 35-44 (d) n=178 | 83 | 87 | 83 | 80 | 82 | 82 | 71 | 70 | 79 | 66 | 56 |
| 45-64 (e) n=315 | 84 | 87^c^ | 83 | 82 | 77 | 77 | 80 | 75 | 70 | 70 | 60 |
| 65+ (f) n=278 | 83 | 80 | 81 | 84 | 86^e^ | 77 | 76 | 72 | 70 | 66 | 54 |
| COVID-19 comorbidities |  |  |  |  |  |  |  |  |  |  |  |
| Yes (g) n=367 | 82 | 85 | 80 | 84 | 79 | 75 | 70 | 69 | 65 | 66 | 51 |
| No (h) n=643 | 83 | 82 | 83 | 80 | 80 | 78 | 78^g^ | 76 | 75^g^ | 69 | 57 |
| Parent/Guardian |  |  |  |  |  |  |  |  |  |  |  |
| Yes (i) n=325 | 90^j^ | 88^j^ | 85 | 85 | 82 | 81 | 78 | 82^j^ | 77 | 73 | 63^j^ |
| No (j) n=688 | 79 | 80 | 81 | 79 | 79 | 75 | 74 | 69 | 69 | 66 | 51 |
| Race/Ethnicity |  |  |  |  |  |  |  |  |  |  |  |
| White (k) n=599 | 82 | 84 | 81 | 80 | 82 | 76 | 76 | 73 | 71 | 66 | 52 |
| Black (l) n=158 | 78 | 77 | 78 | 76 | 76 | 76 | 72 | 62 | 63 | 66 | 63 |
| Hispanic/Latino (m) n=183 | 89^k^ | 84 | 89^k^ | 89^k^ | 82 | 81 | 82 | 82^k^ | 77 | 74 | 65^k^ |
| Annual household income |  |  |  |  |  |  |  |  |  |  |  |
| <$50,000 (n) n=453 | 84 | 80 | 83 | 79 | 80 | 80 | 82^op^ | 77 | 76^p^ | 77^op^ | 59^p^ |
| $50,000-<$100,000 (o) n=305 | 84 | 86 | 85^p^ | 86 | 80 | 79 | 74 | 71 | 72 | 66 | 54 |
| $100,000+ (p) n=248 | 80 | 85 | 75 | 78 | 80 | 71 | 68 | 71 | 65 | 56 | 49 |
| Metropolitan Status |  |  |  |  |  |  |  |  |  |  |  |
| Urban (q) n=323 | 80 | 77 | 77 | 81 | 75 | 74 | 74 | 69 | 69 | 66 | 47 |
| Suburban (r) n=556 | 83 | 87^q^ | 84 | 81 | 81 | 77 | 76 | 75 | 72 | 70 | 57^q^ |
| Rural (s) n=130 | 88 | 79 | 85 | 82 | 84 | 81 | 79 | 75 | 75 | 66 | 65^q^ |
| Education |  |  |  |  |  |  |  |  |  |  |  |
| HS or Less (t) n=389 | 85^v^ | 82 | 85 | 81 | 79 | 83^v^ | 81^v^ | 76 | 79^v^ | 80^uv^ | 63^v^ |
| Some Coll (u) n=272 | 87^v^ | 82 | 81 | 79 | 81 | 80^v^ | 80^v^ | 74 | 76^v^ | 70^v^ | 63^v^ |
| College+ (v) n=356 | 77 | 84 | 79 | 82 | 80 | 69 | 67 | 69 | 61 | 54 | 41 |
| Political Party Affiliation |  |  |  |  |  |  |  |  |  |  |  |
| Republican/leaner (w) n=432 | 93^xy^ | 93^xy^ | 88^y^ | 83 | 86^y^ | 91^xy^ | 88^y^ | 87^xy^ | 87^y^ | 80^y^ | 72^xy^ |
| Independent/unaffiliated (x) n=178 | 80 | 73 | 86^y^ | 80 | 81^y^ | 77^y^ | 81^y^ | 74^y^ | 81^y^ | 75^y^ | 58^y^ |
| Democrat/leaner (y) n=406 | 72 | 75 | 74 | 80 | 72 | 61 | 59 | 57 | 48 | 50 | 33 |
| Religiosity (attendance) |  |  |  |  |  |  |  |  |  |  |  |
| Currently attends services (z) n=360 | 89^A^ | 85 | 88^A^ | 87^A^ | 83 | 82 | 83^A^ | 80^A^ | 79^A^ | 72 | 73^A^ |
| Does not currently attend (A) n=652 | 80 | 81 | 79 | 78 | 78 | 75 | 72 | 70 | 68 | 66 | 46 |

Table notes: Data are from a 2024 nationally representative, probability-based online and telephone survey of 1017 US adults aged 18 years or older. Weighted percentages are displayed. Analyses were conducted using two-tailed *t-*tests. ^a-A^ Value significantly higher than comparison group(s)/row(s) at *P* < 0.05. For race/ethnicity, statistical comparisons were only made between non-White vs. White participants. Full question wording is available earlier in this Appendix.

1. American Association for Public Opinion Research (AAPOR). Best Practices for Survey Research [Internet]; 2022. <https://www.aapor.org/Standards-Ethics/Best-Practices.aspx#best3> [↑](#footnote-ref-1)
2. <https://ssrs.com/opinion-panel/> [↑](#footnote-ref-2)
3. See, for example, Amaya and Presser. Nonresponse Bias for Univariate and Multivariate Estimates of Social Activities and Roles. *Public Opin Q*. 2016; Greenberg and Schneider. Population density: What does it really mean in geographical health studies? *Health Place.* 2023;81:103001; American Association for Public Opinion Research (AAPOR). Data Quality Metrics for Online Samples: Considerations for Study Design and Analysis. November 2022. Available online: https://aapor.org/wp-content/uploads/2023/02/Task-Force-Report-FINAL.pdf [↑](#footnote-ref-3)
4. See, for example, Kohut et al. Assessing the Representativeness of Public Opinion Surveys. Pew Research Center. 2012. http://www.people-press.org/2012/05/15/assessing-the-representativeness-of-public-opinion-surveys; Mercer A, Lau A. Comparing Two Types of Online Survey Samples. Pew Research Center; 2023. <https://www.pewresearch.org/methods/2023/09/07/comparing-two-types-of-online-survey-samples/>; Keeter S, Hatley N, Kennedy C, Lau A. What Low Response Rates Mean for Telephone Surveys. Pew Research Center; 2017. <https://www.pewresearch.org/wp-content/uploads/2017/05/RDD-Non-response-Full-Report.pdf>. [↑](#footnote-ref-4)
5. Sarah Flood, Miriam King, Renae Rodgers, Steven Ruggles, J. Robert Warren, Daniel Backman, Annie Chen, Grace Cooper, Stephanie Richards, Megan Schouweiler and Michael Westberry. IPUMS CPS: Version 11.0 [dataset]. Minneapolis, MN: IPUMS, 2023. https://doi.org/10.18128/D030.V11.0 [↑](#footnote-ref-5)
6. Civically engaged respondents are defined as those who have volunteered in the past 12 months or who talk to their neighbors daily. See the US Census Bureau: https://www.census.gov/programs-surveys/cps/about/supplemental-surveys.html [↑](#footnote-ref-6)
7. See Claritas Pop-Facts Premier: https://environicsanalytics.com/data/demographic/pop-facts-premier [↑](#footnote-ref-7)
8. See Pew Research Centers, https://www.pewresearch.org/methods/fact-sheet/national-public-opinion-reference-survey-npors/ [↑](#footnote-ref-8)
